# Revalorization of Coffee Residues: Advances in the Development of Eco-Friendly Biobased Potential Food Packaging

**DOI:** 10.3390/polym15132823

**Published:** 2023-06-26

**Authors:** Josué D. Hernández-Varela, Dora I. Medina

**Affiliations:** Institute of Advanced Materials for Sustainable Manufacturing, Tecnológico de Monterrey, Monterrey 64849, Nuevo Leon, Mexico; hernandez.varela@tec.mx

**Keywords:** plant-based, biodegradable, compostable, coffee wastes, bioplastics, processing

## Abstract

One of the main limitations in the creation of bioplastics is their large-scale development, referred to as the industrial-scale processing of plastics. For this reason, bioplastic engineering emerges as one of the main objectives of researchers, who are attempting to create not only more environmentally friendly but also sustainable, low-cost, and less polluting materials. This review presents the advances in the development of biodegradable and compostable films/containers using eco-friendly components of by-products of the coffee industry, such as coffee flour (CF), coffee mucilage (CM), coffee husks (CH), coffee silverskin (CS), and spent coffee grounds (SCGs), and a brief review of the common industrial processing techniques for the production of food packaging, including extrusion, compression molding, injection molding, and laboratory-scale techniques such as solvent casting. Finally, this review presents various advances in the area that can be scalable or applicable to different products using by-products generated from the coffee industry, taking into account the limitations and drawbacks of using a biomaterial.

## 1. Introduction

**Figure 1:**
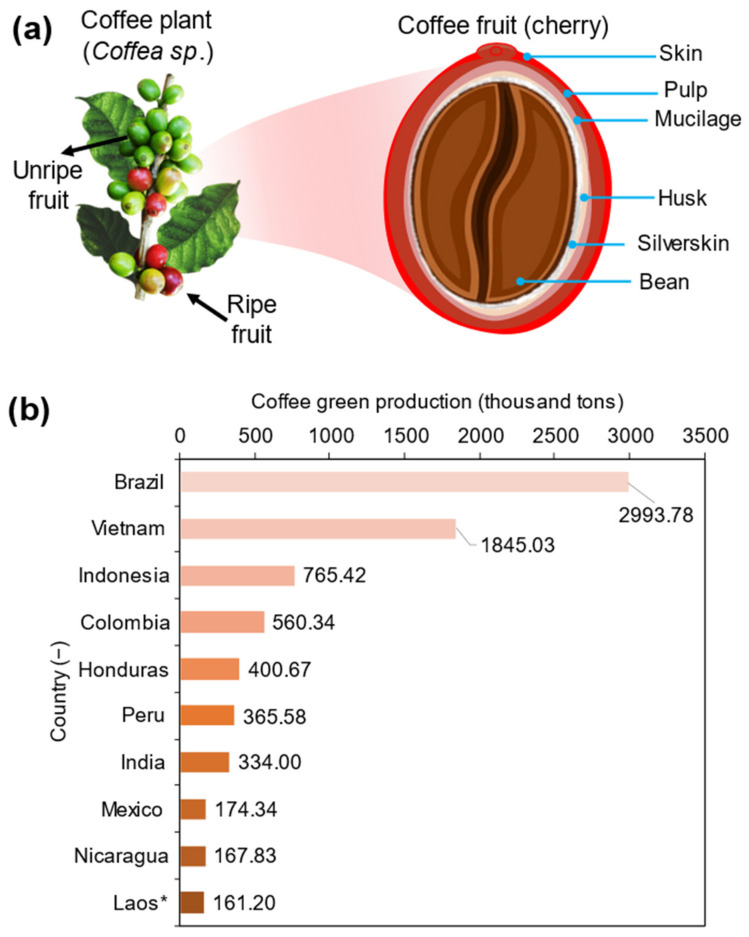
(**a**) Diagrammatic structure of the coffee plant and coffee cherry, with their representative composition. (**b**) Graphic of coffee green production by country, expressed in thousand tons. * People’s Democratic Republic.

Coffee (*Coffea* sp.) is a very popular beverage with a global market that is increasing each day due to the increase in consumption and the growing population [1]. The coffee plant, which is grown in more than 80 countries and follows the petroleum and mining industries, is one of the world’s largest traded commodities [2]. As global green coffee production is increasing by more than 15% per year, important methodological procedures need to be implemented to exploit not only the edible part of the plant but also the residues or wastes from bioproducts [3,4]. Thus, knowing about the main parts of the coffee tree, which is a perennial plant belonging to the family Rubiaceae, is important. The coffee fruit or berry comprises a bright red outer skin (epicarp), which covers the pulp and the highly pectic mucilage (mesocarp). A thin skin layer of endocarp (husk) and the spermoderm layer of silverskin cover the coffee bean [2,5,6] (Figure 1a). Globally, the database for the production of green coffee is provided by the Food and Agriculture Organization [3], and the data offered for an official figure representation of each country for the year 2021 revealed that Brazil is a leader in the production of green coffee, followed by Vietnam and Indonesia (Figure 1b).

Nevertheless, the main edible product from coffee fruit is the coffee beans, which represent less than 50% of the total fruit [2,7]. However, once the beans are thermically processed by dehydration and, later on, ground to obtain spent coffee grounds and extracted with the nutritional components of the bean, the total amount of residues increases to up to 90–95% of the total fruit. This process, known as the brewing process [1], is the most common method to obtain a liquid extract that is rich in caffeine, chlorogenic acid, trigonelline, soluble fiber, proteins, lipids, minerals, niacin, melanoidins, and volatile compounds for a 100 mL brewed coffee [8]. Although this composition is nutritionally important, a huge number of residues are found after this process that are referred to as spent coffee grounds (SCGs), and they are not included in further value-added processing, thus creating a re-used agro-food industry that can be exploited, as the main components of these residues are lignocellulosic compounds [6]. Actually, SCGs are only used in the agricultural industry as compost for farming. However, huge amounts of SCGs become a problem since no technology for processing to avoid mold growth is implemented [9].

This problem of managing the huge amounts of residue generated by the coffee industry is not a concern for producers, but globally, these residues can be possibly used to create biodegradable and compostable packaging that considerably reduces the uses of petroleum-derivate plastics [10]. This strategy is based on the 2030 Agenda for Sustainable Development created by the United Nations to take urgent actions against climate change [11]. Thus, researchers are searching for new strategies to use these agro-industrial and food residues to create biopolymeric materials that can supply the amount of nonrenewable plastic and improve the waste management plan, as the new materials are environmentally friendly and will be included in a circular economy if possible.

Nonetheless, only a few reports using SCG are found in the literature [12,13], although the research on individual residues for all the processing lines in coffee cherries, such as coffee skin and coffee pulp (CP), coffee mucilage (CM), coffee husk (CH), coffee silverskin (CS), and the named SCG, is still unfound in the academic browser. Meanwhile, studies based on the uses of raw or processed residues from the coffee industry are extremely limited and almost difficult to find. Indeed, a quick search of “coffee mucilage” on Scopus produced a large number of related articles, with a total of articles 121 from 1955 to 2023; a peak in these studies was observed in 2021, when 21 research articles were published, but only a few were related to the uses of this residue in new polymeric matrixes, which are the main objective of this review.

For this reason, this review searched alternatives for the re-use and revalorization of residues from coffee fruits (processed or not) to create biopolymeric films with enhanced physical and chemical properties. This review compiles the new advances in the production of bioplastic using coffee residues as a reinforcement material in biobased polymeric films, with further application in food packaging.

## 2. Re-Use and Revalorization of Coffee Residues by Means of Biopolymeric Films Preparations

Some of the common nonrenewable and renewable materials obtained from different sources are known today [14]. Since renewable materials are used and extracted from natural sources such as chitosan, gums, and starches, they have a convenient interaction with the environment and potential applications as biopolymeric plastics worldwide [15]. Until now, chitosan [16] and alginate [17] have been used in several applications as raw components, and coffee residues could increment not only the uses of the product itself but also the by-products due to the main chemical components originating from the material. Nonrenewable materials such as poly(ethylene terephthalate) (PET), polyethylene (PE), polypropylene (PP), and Polystyrene (PS) are continually used due to their excellent characteristics, such as mechanical resistance and chemical inertia [18], but other compounds such as poly(vinyl alcohol) (PVA), poly(lactic acid) (PLA), and poly(1,4-butylene succinate) (PBS) are emerging as biodegradable alternatives which can be used for the development of biopolymeric matrix with reinforced biobased materials [12,19,20].

Nowadays, various methods are commonly used for the development of biobased and ecofriendly packaging films [21,22]. Three techniques are widely used for film preparation (i.e., solution casting, compression molding, and extrusion). Indeed, the casting solution process is a wet process and the easiest way to prepare biopolymer-based packaging films at the laboratory scale [23]. Compared with wet processing, dry processing is a more complicated and industrialized technology that involves the thermoplastic characteristic of biopolymers and the final type of product that is needed [24]. Despite the considerable and potential advantages of dry processing, only a few studies reported the obtention of bioplastic-based materials in biopolymeric films using coffee residues. Table 1 presents the processing techniques and biobased materials used to obtain biopolymers from different sources of coffee residues, and a discussion is presented below for each type of by-product and the technique used to obtain the film.

**Figure 2:**
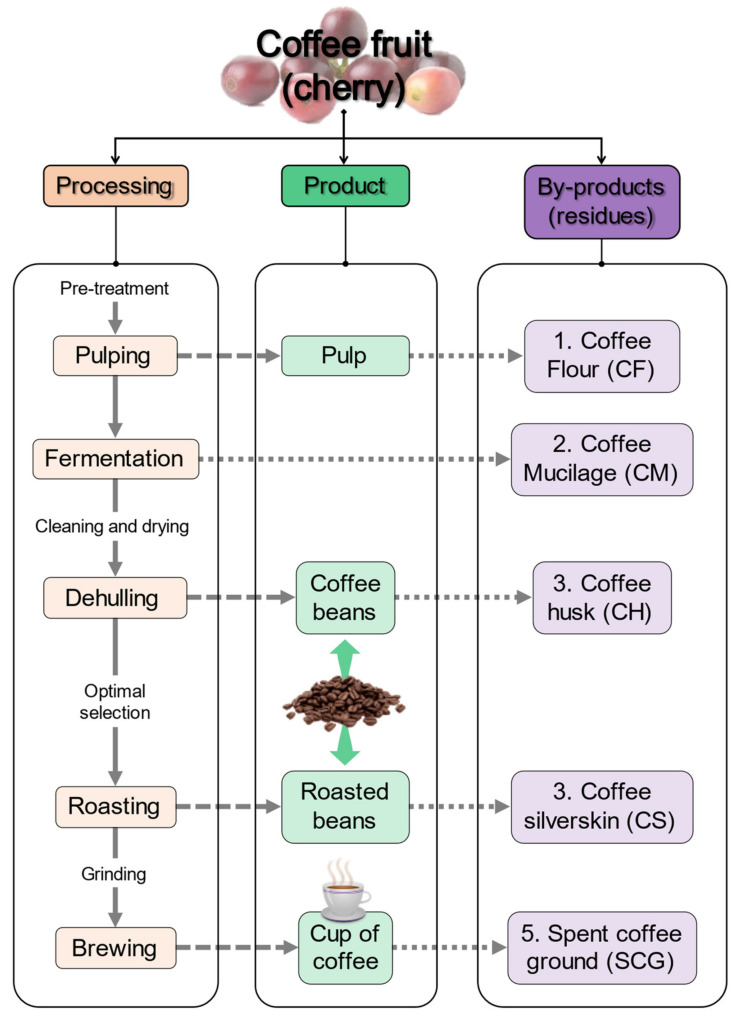
Schematic representation of the processing, products, and by-products generated from coffee cherries.

It is well-known that coffee is considered one of the major edible products that result in the consideration of by-products. Based on the type of processing, product, and by-product (Figure 2), coffee cherries are pretreated to initiate the pulping of the fruit, and the pulp is later transformed into coffee flour (CF), which is the least used residue from coffee cherries. After pulping, the fermentation process releases a small portion of biomaterial known as coffee mucilage (CM), which is rich in pectin. After the fermentation of coffee cherries, a dehulling process occurs, and a thin skin from the coffee cherry is detached to obtain the main product, which is coffee beans, and by-product, which is coffee husk (CH).

After the selection of the best coffee beans by size and morphology, the coffee beans are roasted, and another outer skin is removed from the top part of the bean. This material is the tegument, which directly covers the coffee seed, and it is known as the coffee silverskin (CS) because the color is less brown than coffee beans. In the last processing step, coffee beans are ground to several sizes to obtain coffee grounds, which are mainly used in daily life to prepare coffee, a beverage globally known for its flavor, color, and bioactive compounds [25]. After the brewing of coffee beans, a residue called spent coffee grounds (SCGs) is obtained. This residue accounts for 45–50% of the beans and is considered an accumulative waste with a high lignocellulosic composition [2]. The uses of coffee residues in the formulation of biopolymeric plastics using wet- and dry-processing techniques have been evaluated. Table 1 presents the latest advances in the different types of coffee residues and condenses the major information of the process based on previous studies, which are discussed separately for each residue below.

**Table 1:** Detailed comparison of several bioplastics created from biopolymeric matrix reinforcer with coffee residues (CF, coffee flour or pulp; CM, coffee mucilage; CH, coffee husk; CS, coffee silverskin; SCGs, spent coffee grounds) by different processing techniques (SC, solution casting; ES, electrospinning; C, compression molding; E, extrusion).

Coffee Residue	Extracted Sustainable Component	Supporting Biopolymer	Plasticizer or Compatibilizer	Processing of the Composite	Temperature of Drying	Applications	Reference
Coffee fluor or pulp (CF)	Coffee pulp	Methylcellulose from coffee pulp	Polyethylene glycol—1% *v*/*v*	SC—glass plates	60 °C by 25 min Room T for 2 days	Vehicle for incorporating additives in food packaging	[26]
	Coffee pulp extract	Chitosan	Glycerol—0.90% wt.	SC—Teflon plates	30° and 40 °C 25 °C by 2 days	Reduction of the microbial contamination in food products	[27]
	Pectin and cellulose from coffee pulp	Pectin and microcrystalline cellulose from coffee pulp	Glycerol—20% (*w*/*w*) from biopolymers	SC—Petri dishes	45 °C by 16 h	Re-uses of agricultural biomass in food and packaging industries	[28]
Coffee mucilage (CM)	Pectin from CM	Pectin from CM	Glycerol—22% pectin weight Tween 20—1:10 pectin	SC—rectangular acrylic mold ES—syringe for the injection of and a rotary drum as the collector	30 °C by 8 h	Feasible films and coatings for food	[29]
Coffee husk (CH)	Fibers from CH	High-density PE, maleate PE, wood flour	None	E—twin/single screw	Melting: 160 °C Pumping: 170 °C Die: 170 °C Twin screw: 40 rpm Single screw: 20 rpm	Environmentally friendly alternative for preparation of polymeric composites.	[30]
	Antioxidant extract and cellulose from CH	Starch, poly (e-caprolactone), PLA	Glycerol—30% starch weight.	C—hot-plate press with Teflon sheets	160 °C by 1 min at 30 bars, followed by 3 min at 130 bars, 3 min cooling cycle	Reduction of light-induced oxidation reactions in food-packaging applications	[31]
	Dried CH	Starch	Glycerol—30% starch weight	SC—PS Petri dishes	45 °C by 24 h	Promising alternatives in sustainable food packaging	[32]
Coffee silverskin (CS)	Cellulose nanocrystals from CS	PLA	None	E—co-rotating twin-screw with sheet die	Feeder: 170 °C Die: 180 °C Screw: 150 rpm	Limited application in industrial food packaging and medical devices	[33]
	CS functionalized with Alkaline and Palmitoyl-chloride	High-density PE	Maleic anhydride, dicumyl peroxide (reaction initiator)	E—co-rotating screw extruder Micro Injection Molding Machine	Melting: 150 °C Pumping: 160 °C Die: 170 °C Screw: 50 rpm	Reliable applications in aerospace, automotive, and packaging.	[34]
	Milled CS	PLA, PBS	None	E—twin-screw with film casting	Melting: 160 °C Die: 185 °C Screw: 120 rpm	Production of degradable packaging materials	[4]
Spent coffee grounds (SCGs)	Chloroform-based extract from SCGs	PLA, Diatom	Chloroform	SC—Teflon Petri dishes	Fume hood up to 72 h	Enhance mechanical and oxygen barriers for food packaging applications	[35]
	Milled SCGs	Corn starch	Glycerin	SC—polyacrylic molds	Oven at 32 °C, 4 days	Alternative substitute for some types of packages	[36]
	TEMPO-oxidized cellulose nanofibers from SCG	PVA	None	SC—Teflon square dish	40 °C overnight in vacuo	Huge number of possibilities due to the compatibility of the composite film	[37]
	Ball-milled SCG	PLA	None	(1) E—twin screw (2) E—single screw (3) E—blow film	(1) 120 °C in feeding and 180 °C in the die, at 100 rpm (2) From 140 to 180 ºC at 65 rpm (3) Roll speed at 2.3 m/min	Biocomposite films for degradable plastic bags	[12]
	Macerated organic SCG	PLA	Functionalized oligomers of lactic acid with maleic anhydride	(1) E—twin-screw (2) Injection molding	(1) From hopper to die: 180–185–190–195 °C at 22 rpm (2) 185 ° C (hopper) and 200 °C (injection nozzle) with a clamping force of 75 tons	Promising disposable food-serving utensils and tableware	[38]
	Antioxidants from SCG	Whey-protein concentrate	Glycerol—40% from supported biopolymer	SC—Teflon dishes	25 ± 2 °C for 24 h, peeled off and conditioned for 48 h with magnesium nitrate	Preparation of edible films with a fundamental circular economy	[39]

### 2.1. Coffee Flour Residues

The first by-product obtained from coffee is the coffee flour (CF), which is the result of cleaning and removing the first layer of the coffee cherries. The main content is known as coffee pulp (approx. 43% of the weight of the coffee fruit), and this nonedible part of the cherry is identified when coffee seeds are extracted [26]. The pulp is a hydrated part of the coffee cherry since water is used in the remotion of it, producing an increase in the moisture content (78–81% wt.) [26,27,28]. After drying, the CF is obtained and mostly composed for cellulose (36%), pectic polysaccharides (21%), hemicelluloses (9%), and free sugars (5%) [40,41]. Even when coffee pulp can be transformed into CF by mechanical treatments, there are only a few reports presented in the literature with the uses of this by-product in the formulation of biopolymeric films.

In this sense, Venkatahalapathy and Adwitiya [26] used coffee pulp to obtain a methylcellulose material from the by-product after a series of chemical isolation processes. The development of this functionalized methylcellulose was exploited for the preparation of packaging materials with enhanced antioxidant and mechanical properties; the authors formulated a film which includes 85.19% of natural cellulose, 14.81% coffee pulp methyl cellulose, and 18.5 g of antioxidants (extracted from coffee pulp), which improved the antioxidant capacity of the developed films. In addition, some mechanical properties (such as tensile strength) of the film increased, and they found that the developed film is similar to those prepared with the control PP. These results can be used as parameters to create films that act as supports and vehicles for the incorporation of additives in food packaging. As some authors have previously reported, antioxidants can be added to films as raw composites from the coffee pulp, and some authors studied the behavior of a water-based extract from raw coffee pulp and its addition to a chitosan-supporting biopolymer. Jaisan and Punbusayakul [27] prepared four different chitosan-based films with varying concentrations of coffee pulp extract, and they observed an increased tendency in the scavenging activity when a 2,2-diphenyl-1-picryl-hydrazyl-hydrate assay was conducted. Furthermore, the water vapor and solubility behavior of the film showed decreased values due to the increasing of hydrophobic group when the extract was added. Specifically, the formation of hydrogen bonds between the -NH2 group presented in a -CH and -OH group of the phenolic compounds is responsible for this behavior. These results are promising for the use of these films in food products and help avoid important problems such as microbial contamination.

However, the antioxidant activity observed from coffee pulp is not reliable, as high amounts of residues are needed to decrease landfill contamination; thus, other techniques must be implemented to use coffee residues. Dao et al. [28] exploited the uses of the major biopolymers found in coffee pulp (pectin and cellulose) to produce biopolymer films. Given that these compounds are trapped in a complex matrix, two steps were used to isolate pectin and cellulose. For pectin, a step using an acidified solvent at a high temperature recovered 20% pectin. For cellulose, the first step was a pretreatment using vapor, and the second step was the application of a more ecofriendly bleaching step to extract cellulose. The authors claimed that the citric acid/water solution for the extraction of pectin is a novel method in comparison with those presented in the literature [42,43]. In addition, they hypothesized that microcrystalline cellulose plays a reinforcing role in film formulation, which produces a smooth surface, high transparency, and better mechanical properties in the final films. 

### 2.2. Coffee Mucilage Residues

Since coffee pulp is considered a product, and its by-product is the CF, which is the dry part of the pulp, the pretreated cherries are exposed to a mechanical or enzymatic process when coffee beans are placed in fermentation tanks [6]. In this process, an inner layer, sticky and strongly adhered to the coffee green bean, known as coffee mucilage (CM), is found and needs to be removed during the process in order to obtain a cleaner green bean before the dehulling process (Figure 2). Even when large amounts of CM are found in the production of clean green beans (around 22% wt.), only a portion of this by-product is used in the production of new biomaterials. CM has significant concentrations of cellulose [44,45], pectic substances [29], and neutral non-cellulosic polysaccharides [46], but only few studies reported the obtention of re-valuable products from this residue.

However, the significant presence of pectic polysaccharides (>30%) reveals that CM has a remarkably high potential for the development of bioplastics using the extracted compound to enhance some food-packaging properties. Particularly, electrospraying (ES) emerged as a novel technique for the preparation of biodegradable films based on pectin from CM, as an alternative to reduce the environmental impact of some residues from the coffee industry [29]. Since CM has a polymeric relationship that is characteristic for this pectin-based compound [46], some of its mechanical and barrier characteristics are indicative of a typical behavior of rigid polymers. However, the water-resistance properties indicate that this type of new biopolymeric film has a highly biodegradable character because of its solubility in water [29]. Indeed, the authors informed that the composition of pectins from citric and CM differs, and the different processing methods promoted improvements for several properties, such as water vapor permeability, solubility, and biodegradability. This conducted study reveals the unexploited opportunities of CM, using a mixture of experimental techniques.

Regarding the method of film preparation used in an investigation [29], a realistic comparison was performed between an emergent and a more common technique. Based on the principle of ES, which involves an electric potential difference and is applied to liquid–air surfaces, a comparison with the more useful solvent-casting method was made. The results indicated that electrospraying created uniform coatings with large surface areas, and this is contradictory to the finding obtained with the casting method, which uses higher volumes of raw material, has more time-consuming electrical equipment, and requires more efforts to obtain reproducible films [47]. Up until the publication of this review, no further studies using CM were performed or published.

### 2.3. Coffee-Husk Residues

The next by-product found in the inline obtention of coffee beans before roasting is the coffee husk (CH). This residue is considered a very valuable waste in the dry processing of coffee beans, as the wet processing delivers a better coffee grain, but the residues need more treatment [7]. For the removal of CH, the outer skin is obtained through the drying and de-husking of coffee cherries, and the process is considered an aggressive technology, as the material is thick and hard compared with other parts of coffee cherries [2,5]. However, the main idea for the revalorization of CH is its use as a filler material since the biomaterial has a porosity of approximately 80%, which means that it occupies large spaces but has a very low weight [48]. Moreover, the increased production of CH, which is up to 30–50% of the dry basis of coffee cherries [49], could be exploited to reduce landfill contamination.

Nevertheless, the chemical composition of CH, which has been studied and compilated as cellulose, hemicellulose, lignin, and ashes [30,50,51], decreases the opportunities of using these residues in their raw form. Indeed, CH needs to be pretreated with or accompanied by compatibilizers to be included in the formulation of biopolymeric films. This condition is due to the lack of free hydroxyl groups to react with other polymers which creates a low interfacial adhesion between the matrix components [30,51]. In order to increase the interfacial adhesion of these materials, different compounds, such as citric acid, stearic acid, maleic anhydride, dicumyl peroxide, or citrate esters, have been used in the preparation of blends [52]. These chemical reactants have been used to enhance blend preparations, as well as the mechanical, thermal, and barrier properties of the final products, and some examples are presented below.

Recent studies using isolated fibers from CH revealed a very important interrelation between high-density PE and maleate polyethylene when wood flour is used as a filler composite [30]. For this processing stage, the extrusion (E) method, using a twin/single screw and three different temperatures, was applied in the extrusion machine. The elevated temperature and the velocity of the screws (40 rpm for the twin screw and 20 rpm for the single screw) were common extrusion parameters used for dispersing additives in the concentrate and minimizing the problems associated with self-wiping capability and conveying [53]. Upstanding results were found, with 40 to 70% loading of CH resulting in an increase in the modulus and thermal properties of the composite. Moreover, when maleate polyethylene was added to the mixture as a coupling agent, a very significant interfacial behavior was achieved, and it induced a better interaction between the hydrophilic lignocellulosic fiber and the hydrophobic polymer matrix. Given that PE is considered a nonrenewable and nonbiodegradable material, this process can create an environmentally friendly alternative for the preparation of polymeric composites, minimize the bioaccumulation problem, and enhance high-water resistance in biopolymeric materials.

Although extrusion can be referred to as an excellent processing of bioplastics, other processes, such as compression molding, can significantly enhance film properties when the right process is performed. Collazo-Bigliardi et al. [31] used CH to obtain antioxidants, cellulose fibers, and cellulose nanocrystals, and after the preparation processes, different amounts of these by-products were included in the poly (e-caprolactone) and PLA film, using glycerol as a plasticizer. The results indicated that the reinforcing tensile properties of the material were improved (from 45 to 515 MPa) by the inclusion of cellulose nanocrystals (aspect ratio between 20 and 40 nm), and this finding may be due to their incorporation into the polymeric matrix as a supporting material [10]. However, the antioxidant extract was a filler component with low mechanical performance in the blend films; regardless, the authors confirmed that antioxidants have a special character to reduce oxygen permeability, and this behavior prevents light-induced oxidation reactions in food-packaging applications.

Finally, a comparable methodology that uses glycerol as a plasticizer but with a solvent casting method was applied. Schutz et al. [32] developed a starch film by using dried CH after a sieving process in a starch/glycerol blend. Promising results on alternatives in sustainable food packaging were reached as a certain concentration of CH was incorporated into the film, providing an effective barrier against ultraviolet light and conferring thermal stability and mechanical properties to the films. The evaluation of the UV barrier was calculated to be in the range of 200–350 nm, since this can rapidly cause oxidative degradation of foods, becoming a bigger problem in storage. The films with CH presented a transmittance value close to zero, revealing an important protection against UV light when the reinforced agent is added. Since it was explained before, the incorporation of this lignocellulosic material provided a more hydrophobic matrix that avoided water permeability and absorption, which is an excellent quality for this type of biopolymeric film with applications in food packaging.

### 2.4. Coffee Silverskin Residues

Prior to brewing, coffee beans are roasted to enhance their distinct aromas, taste, and color [54]. This roasting process releases a thin layer, namely coffee silverskin (CS), which is a tegument of green coffee beans and represents approximately 4% (*w*/*w*) of coffee beans [13]. The main idea for the roasting process is to preserve the composition of volatile compounds in the coffee beans, such as melanoidins, which are formed through Maillard reactions during roasting and have been investigated as nitrogenous high-molecular-weight heterogeneous polymers [55,56]. The processed coffee beans exhibit organoleptic changes that are predominated by modified phenolic substances which are present naturally in coffee beans but affected by the duration and temperature of the process [5]. However, few reports on the applications of CS in film production are found, although their composition consists mainly of cellulose, hemicellulose, lignin, and phenolic compounds, which can be used in dietary applications [13,57,58].

However, the processing of these biomaterials has increased their application in polymeric materials by highly technological techniques (such as extrusion) since the wastes only need to be ground and sieved before being used as a filler or reinforcing supporting material in the final composite [4]. Nevertheless, previous reports indicated the usage of PLA in the formulation of a biopolymeric blends with cellulose nanocrystals extracted from CS by means of an alkali and bleaching treatment [33]. In this process, an industrial procedure is considered to obtain biopolymeric blends via the extrusion technique, mixing the cellulose nanocrystal with PLA in different ratios (1%, 3%, and 5% of nanomaterial). The results indicate that the tensile strength and Young’s modulus increased by increasing the addition of the nanomaterial, and the water vapor permeability decreased gradually by increasing the addition of CNCs. These results are promising since cellulose nanocrystal acts as a reinforcing material in the good oxygen barrier properties, as well as the mechanical properties, of the films, revealing that reproducible and well-made materials can be implemented in diverse applications not only for industrial food packaging but also for medical devices [33,59]. Further advantages will be found, as solvents and compatibilizer were not used in this investigation, thus providing an opportunity to create a more environmentally friendly and economically undesirable method that can be applied more frequently.

Recent studies used functionalized CS and compatibilizers to obtain reliable applications in aerospace, automotive, and packaging. In this case, Dominici et al. [34] employed an process involving alkali bleaching and esterification with palmitoyl chloride to functionalize CS and, later on, created a polymeric blend using high-density PE by means of extrusion. These functionalization processes reveal an additional term for biopolymeric films based on non-degradable materials which is known as “bio-based PE”, using the physical technique to create a material more degradable than only PE [60]. Interesting results were obtained when maleic anhydride and dicumyl peroxide were used (as compatibilizer and reaction initiator, respectively) in the formulation of the composite. Based on their mechanical, thermal, morphological, and water-absorption behaviors, the produced films (90 µm in thickness) by means of a micro-injection molding machine showed that this agricultural waste could be transformed into a more affordable, renewable, and ecofriendly material. Finally, a rough experiment using milled CS in the polymeric preparation using mixtures of PLA and PBS revealed an increase and acceptable mechanical properties based on the different additives used [4]. The most outstanding results are based on the usage of CS as bulk and thin-film material. The first processing material showed an improvement in mechanical properties (increasing elastic modulus and decreasing strain from 1500 to 2500 MPA and from 9 to 3%, respectively), adhesion with the polymeric matrix, ductility in films, and critically reduced crystallinity values. Second, when functionalized CS was added to the production of thin films, a substantial reduction in the water uptake was obtained (around 13%). The processing of polymers using maleated PE reduced the biggest amounts of OH groups and hydrophobized the CS, which is one of the most outstanding results for further processing techniques. These results could be employed as drug delivery or cell delivery, acting as an alternative as chitosan has been in the last decade [61,62].

Although the biopolymeric film was produced by means of a twin-screw extruder and film solvent casting, other trials are needed to evaluate the promising results related to biodegradability. The authors claimed that composting and disposability profiles need to be determined to provide an acceptable result in terms of the environmental impact.

### 2.5. Spent Coffee Ground Residues

The last by-product obtained from coffee beans relies on a process that involves the brewing of milled coffee beans to obtain the most consumable part of the coffee industry: the coffee beverage [2]. This process generates a massive number of residues known as spent coffee grounds (SCGs), which are deposited in the landfill and degrade naturally to incorporate some compounds into the crop. However, new investigations revealed the high potential of SCGs for use in industries such as bio-syngas, biodiesel, green composites, and soil amendment [63]. This condition is due to the high amount of residual waste obtained, which is estimated to be approximately 90% of brewed coffee. In total, SCGs are considered to be the most quantifiable by-product of the coffee industry, and 5–10% of coffee is consumed as a drink globally. For a specific sum of by-products, the CF, CM, CH, and CS account for 45–50% of the total coffee cherry, and the values for the brewed coffee and SCGs reach to 5–10% and 45–50%, respectively, depending on the extraction method [2,6,63].

Several studies reported the re-use of this biomaterial. Gazonato et al. [36] used a milled SCG mixed with cornstarch and glycerin as the plasticizer. The method film preparation by solvent casting is a very low-cost and friendly process when biopolymers are used, but the duration of the film drying can be extended in some cases, causing the process to be expensive or less sustainable. Changing the processing method of biopolymers can signify a more extensive and time-consuming process when different extrusion techniques are used. Suaduang et al. [12] ball-milled the processed SCGs with PLA in three different extrusion ways: twin screw, single screw, and blow film. The results indicate that the mechanical properties were reduced when the SCG content was increased (values between 276 and 394 were found at 10%SCG). Similar to this finding, the higher contents of SCGs changed the behavior of the melt flow rates and flowability. This investigation revealed another promising methodology for the usage of SCGs as a filler in biopolymeric blends with an important resolution of the contamination problem by by-products and increased added value of this important biological waste product. Similar to the work of Suaduang et al. [12], the work by Terroba-Delicado et al. [38] included macerated organic SCGs with PLA in a blend, using a twin-crew extruder and injection molding to obtain the final blend. For the compatibilizer, some functionalized oligomers of lactic acid and maleic anhydride were used, and the results indicated that the addition of these compounds contributed substantially to improving the tensile strength of the composites (up to 60%). In addition, the work demonstrated a feasible and reliable method to manufacture PLA-based green composites using residues from the coffee industry through the revalorization of wastes.

On the other hand, other valuable products, such as cellulose nanofiber and antioxidant extracts, can be used in the formulation of films by solvent casting. Essentially, Kanai et al. [37] isolated the cellulose form of SCGs via catalytic oxidation, using the TEMPO method. In this process, cellulose can be transformed into nanocellulose via the oxidization of hydroxyl groups into sodium carboxylate groups in the surface of microfibrils. This nanomaterial can be disintegrated easily in water by mechanical stirring and used later in the preparation of water-based biopolymeric films. Furthermore, PVA acted as a supporting polymer, and the results defined the TEMPO-oxidated cellulose from SCGs as an alternative source of wood-derived nanocellulose with a high compatibility for the creation of composite films by solvent casting.

Finally, another way to incorporate residues from SCGs into the formulation of films is through the use of organic-based extracts obtained by simple solvent extraction, as reported by Cacciotti et al. [35] and Papadaki et al. [39]. In both cases, solvent casting was used to obtain biopolymeric films, and distinctive advantages were found. Cacciotti et al. [35] used PLA and calcined diatomaceous earth as supporting polymers. The results indicated that the addition of a chloroform extract from SCGs to the prepared film noticeably enhanced the mechanical and oxygen barriers of the film (values are 500–800 MPa and 53 cm^3^·mm/m^2^·day, respectively), which are good qualities for food packaging applications. In addition, Papadaki et al. [39] incorporated an antioxidant extract from SCGs into a whey-protein concentrate film plasticated with glycerol, providing a new way to create edible films with a fundamental circular economy that improves the uses of residues with more sustainable and green chemistry pathways.

## 3. Future Perspectives

To date, the advances in the use of 0-based products, such as those derived from the coffee industry, can considerably reduce the environmental footprint produced by plastics derived from petroleum. However, a latent problem is the biodegradability, compostability, and/or recycling process when non degradable polymers are used. Thus, researchers are studying new (or improved) methods for using functionalized coffee residues to make polymeric composites to be exploited on a major scale given that the design and production of effective eco-plastics must focus on material composition, their degradation, and degradation rate, regardless of whether they will end up in the recommended ecosystem. However, studies and results on different composites obtained in the literature may vary according to the waste management standards of each country or society; indeed, the new regulation based on the amount of biobased material into the polymeric matrix needs to be reconsidered in order to evaluated if the production of biobased composites can be a reality. Therefore, the knowledge gap must be filled, and the political gap must be closed, as non-degradable materials are a problem that affect everything, and everyone can contribute different ideas based on the effectiveness of biobased plastic decomposition in various environments and the possible use of other compatibilizers that are more friendly to the environment.

## 4. Conclusions

This review offers an overview for potential biobased and environmentally friendly food packaging by means of the most common film production processes using coffee residues. Since agro-wastes are emerging as a competitive bio-sourced raw materials, several alternatives need to be implemented to achieve high yields of the new biobased materials with further packaging applications. The outlook provided by this review is that plastics formulated and/or reinforced with residues from the coffee industry (such as CH, CS, and SCG, which are the most studied ones, even though all coffee by-products have the potential for this purpose) need to be exploited on a macroscale to revolutionize not only the production of plastics but also the uses and advantages that can be contemplated in the future.

## Data Availability

Not applicable.
